# Unblocking the rate‐limiting step of the municipal sludge anaerobic digestion

**DOI:** 10.1002/wer.10793

**Published:** 2022-10-02

**Authors:** Jiefu Wang, Yuepeng Sun, Dian Zhang, Tom Broderick, Mary Strawn, Hari Santha, Karen Pallansch, Allison Deines, Zhi‐Wu Wang

**Affiliations:** ^1^ Department of Biological Systems Engineering Virginia Tech Blacksburg Virginia USA; ^2^ Arlington County Water Pollution Control Bureau Arlington Virignia USA; ^3^ Alexandria Renew Enterprises Alexandria Virginia USA; ^4^ Stantec Fairfax Virginia USA

**Keywords:** anaerobic, hydrolysis, mesophilic, sludge, SRT, thermophilic

## Abstract

Anaerobic digestion stabilizes municipal sludge through total solids reduction and biogas production. It is generally accepted that hydrolysis accounts for the rate‐limiting step of municipal sludge anaerobic digestion, impacting the overall rates of solids reduction and methane production. Technically, the sludge hydrolysis rate can be enhanced by the application of thermal hydrolysis pretreatment (THP) and is also affected by the total solids concentration, temperature, and solids retention time used in the anaerobic digestion. This study systematically analyzed and compared ways to take these four factors into the consideration of modern anaerobic digestion system for achieving the maximum solid reduction. Results showed that thermophilic anaerobic digestion was superior to mesophilic anaerobic digestion in terms of solids reduction but vice versa in terms of the methane production when integrated with THP. This difference has to do with the intermediate product accumulation and inhibition when hydrolysis outpaced methanogenesis in THP‐enhanced thermophilic anaerobic digestion, which can be mitigated by adjusting the solids retention time.

**Practitioner points:**

- THP followed by TAD offers the greatest solids reduction rate.
- THP followed by MAD offered the greatest methane production rate.
- FAN inhibition appears to be an ultimate limiting factor constraining the methane production rate.
- In situ ammonia removal technique should be developed to further unblock the rate‐limiting step.

## Graphical abstract

Four factors are taken into the consideration for achieving the maximum solid reduction.

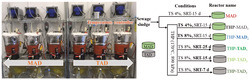

## INTRODUCTION

Anaerobic digestion is a biological process commonly used in water resources recovery facilities (WRRFs) for reducing the mass and volume of the primary and secondary sludge produced during wastewater treatment (Mills et al., 2014) and, in turn, saves the cost of biosolids transportation and disposal in the landfills or on the farmlands. Along with this solid reduction, the organic fraction of the sludge also can be biologically converted to biogas that can be utilized as a type of renewable energy for generating heat, electricity, and even vehicle fuel (Tian et al., 2021). Similar to the solids fermentation processes used in other fields (Chilakamarry et al., 2022; Liu et al., 2020), the rate of solids hydrolysis is usually the limiting step constraining the overall rate of the anaerobic digestion processes consisting of hydrolysis, acidogenesis, acetogenesis, and methanogenesis (Appels et al., 2008; Wang et al., 2011). Therefore, the purpose of this study is to develop a strategy that can substantially improve the municipal sludge hydrolysis rate, which is highly desired in industrial practices to unblock this rate‐limiting step for upgrading the capacity of existing anaerobic digesters.

Technically, the sludge hydrolysis rate is affected by the total solids concentration (TS) (Di Capua et al., 2020; Xu et al., 2021), the solids retention time (SRT) (Appels et al., 2008; Ge et al., 2011; Jain et al., 2015; Pilli et al., 2015; Zhen et al., 2017), and the temperature (e.g., mesophilic or thermophilic) used in anaerobic digestion (AD) processes (Müller, 2000). In recent years, thermal hydrolysis pretreatment (THP) has also been increasingly used to further enhance the sludge hydrolysis rate (Appels et al., 2008; Ge et al., 2011; Jain et al., 2015; Pilli et al., 2015; Zhen et al., 2017). To date, these measures have not been investigated in a systematic manner to explore the extent to which the sludge hydrolysis rate can be maximized through the process integration and optimization. To this end, this study is aimed to determine the enhancement these four measures can possibly bring to the municipal sludge AD. It is anticipated that the outcomes from this study are able to provide engineering guidance for WRRFs to maximize the capacity of existing anaerobic digesters.

## MATERIALS AND METHODS

### THP setup

**FIGURE 1:**
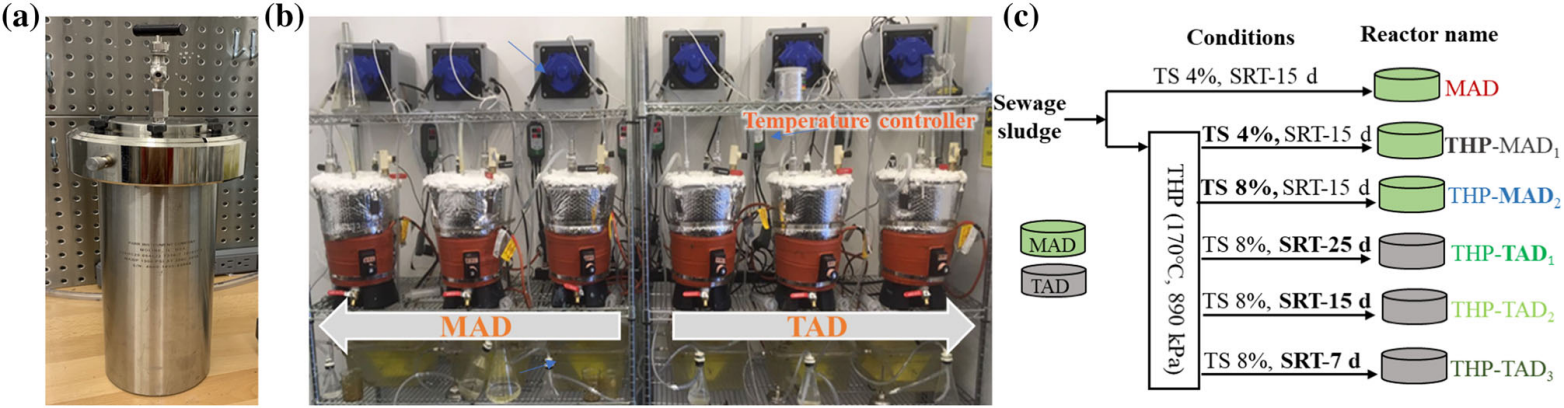
Experimental design: (a) thermal hydrolysis pretreatment (THP); (b) anaerobic digesters; (c) summary of experimental design

THP was performed in a 2 L pressure vessel (No. 4602, Parr Instrument, Moline, IL) (Figure 1a) heated in a muffle furnace at 170°C and 890 kPa for 2.5 h to ensure sufficient heat penetration. The THP temperature was selected based on the specification of full‐scale Cambi THP process (Appels et al., 2008), which was also suggested by previous studies (Di Capua et al., 2020; Zhang et al., 2021) in terms of the extents of sludge solubilization and subsequent biogas production to be achieved under such conditions. We are aware that this lab‐scale THP time (2.5 h) is much longer than the 0.5 h commonly used in full‐scale THP, which uses hot stream for quick heat penetration. Due to the lab‐scale setup limitation, we chose to make this necessary compromise as was practiced in many other similar lab‐scale studies (Zhang et al., 2021). As a result, the outcome from this study is expected to overestimate the THP effects. The sludge processed in this lab‐scale THP was then fed to the lab‐scale anaerobic digesters shown in Figure 1b. The feed sludge characteristics before and after THP is shown in Table 1. More detailed information about the lab‐scale THP setup can be found in the study by Zhang et al. (2021).

**TABLE 1:** Feed sludge characteristics before and after THP

	Raw sludge	After THP
TS (wt%)	4.0 ± 0.1	4.0 ± 0.1
VS (wt%)	78.0 ± 0.58	78.0 ± 0.80
tCOD (mg L^−1^)	41,843 ± 488	42,303 ± 1464
sCOD (mg L^−1^)	3494 ± 81	9074 ± 117
Alkalinity (mg CaCO_3_ L^−1^)	742 ± 19	1419 ± 21
VFAs (mg Acetate L^−1^)	987 ± 49	2496 ± 77
Soluble protein (mg L^−1^)	1671 ± 37	2678 ± 62
Soluble carbohydrate (mg L^−1^)	424 ± 50	3779 ± 7
pH	7.03 ± 0.0	7.11 ± 0.0

Abbreviations: sCOD, soluble chemical oxygen demand; tCOD, total chemical oxygen demand; THP, thermal hydrolysis pretreatment; TS, total solids concentration; VFAs, volatile fatty acid; VS, volatile solids concentration.

### Anaerobic digester setup

A total of six stainless steel anaerobic digesters with the design described in a previous study were employed in this research (Zhang et al., 2021). Referring to Figure 1b, each digester came with a working volume of 5 L was completely mixed via biogas recirculation at a flow rate of 0.5 L min^−1^ from the headspace to the conical bottom. The temperature of these digesters was maintained with insulation layers and heating blankets (50‐425F, Cole‐Parmer, Vernon Hills, IL) via feedback temperature controllers (ITC‐308, Inkbird Tech, Shenzhen, China). The biogas production was monitored using calibrated tipping‐bucket meters coupled with automatic data loggers (HOBO Pendant®, Bourne, MA Archae Press, Nashville, TN). The mesophilic digesters were inoculated with digestate from a full‐scale mesophilic anaerobic digester (MAD) in a local WRRF, and the thermophilic digesters were inoculated with digestate from a lab‐scale thermophilic digester that has been stabilized for 280 days (Zhang et al., 2021). The dewatered sludge cake fed into these digesters was collected from another local WRRF and contains 20.0% TS made of primary and secondary sludge blended in a dry mass ratio of 31:19. Distilled water was used to dilute the cake to 4% or 8% TS according to the experimental design in Figure 1c. Briefly, three digesters were operated under mesophilic (35 ± 0.3°C) conditions, whereas the other three were operated under thermophilic (55 ± 0.3°C) conditions. A one‐factor‐at‐a‐time approach was taken to understand the effect of a factor and then integrate the better level of the factor for the next factor optimization. Therefore, a single variable experiment was designed to evaluate the contribution of each of the four factors to the hydrolysis rate enhancement and methane production. Briefly, in order to evaluate the effect of THP on hydrolysis rate, sludges with and without THP were fed into two identical MADs to compare their digestibility. Likewise, the post THP sludge with 4.0% and 8.0% TS were fed into two identical MADs, respectively, to understand the TS effect. A thermophilic anaerobic digester (TAD) was also compared with an MAD to understand the digester temperature effect. In the end, three TADs operated with the SRTs of 7, 15, and 25 days were also operated in parallel to understand the SRT effect (Figure 1c).

### Chemical analysis

Gas samples from the headspace of the digesters were measured for methane contents, using a gas chromatograph equipped with a thermal conductivity detector and a flame photometric detector (Shimadzu, Columbia, MD). The pH, TS, and volatile solids concentration (VS) were analyzed according to the standard methods (APHA, 2012). Alkalinity, volatile fatty acid (VFA) concentrations, and VFA‐to‐alkalinity ratios were quantified using an auto‐titrator (Hanna Instruments, Woonsocket, RI). Sludge samples were filtered through a 0.45 μm PTFE filter (MilliporeSigma, Burlington, MA) to measure the total ammonia, soluble chemical oxygen demand (sCOD), a well as protein and carbohydrate contents using Hach test kits of TNT 832, Hach TNT plus Vial, modified Lowry protein assay kits (Pierce™, Rockford, IL), and the phenol‐sulfuric acid method (Nielsen, 2010), respectively. Total chemical oxygen demand (tCOD) was measured using Hach TNT plus Vial according to the standard methods (APHA, 2012). The free ammonia nitrogen (FAN) concentration was calculated using the ideal equilibrium equation from the study by Emerson, et al. (Emerson et al., 1975) The volatile solid reduction (VSR) ratios were calculated using the equation from the study by Mei et al. (2016). The volumetric methane production rates (L L^−1^ d^−1^) were calculated by dividing daily methane production volume by the working volume of the digester. The volumetric solids reduction rate (g L^−1^ d^−1^) was calculated by dividing daily mass of solids destroyed by the working volume of the digester.

### Statistical analysis

Microsoft Excel was used to tabulate data and perform simple statistics such as mean, standard deviation, and *T* test. *T* tests were used to determine if the mean difference between two variables was statistically significant (*p* < 0.05). The AD steady states were also determined by *T* tests of random monitoring data sampled from the daily pH and volumetric methane production rate profiles presented in Figure 3; that is, experimental data collected after the time behind the dash lines in Figure 3 are with *p* > 0.05 and thus can be regarded as steady state data. The data from THP‐TAD_3_ in Figure 1c did not have a steady state because of the failure in methane production.

## RESULTS

### Effects of THP on sludge hydrolysis

The effect of 170°C THP for 2.5 h on sludge hydrolysis was studied in a MAD operated at an SRT of 15 days. Table 1 shows that the THP itself did not change TS, VS, pH, and tCOD of the sludge. However, THP has substantially increased all the soluble metrics. For example, sCOD, alkalinity, VFAs, and soluble protein and carbohydrate concentrations have increased for 0.6‐ to 7.9‐folds as a result of THP (Table 1), which indicates the effectiveness of THP in hydrolyzing the municipal sludge solids.

**FIGURE 2:**
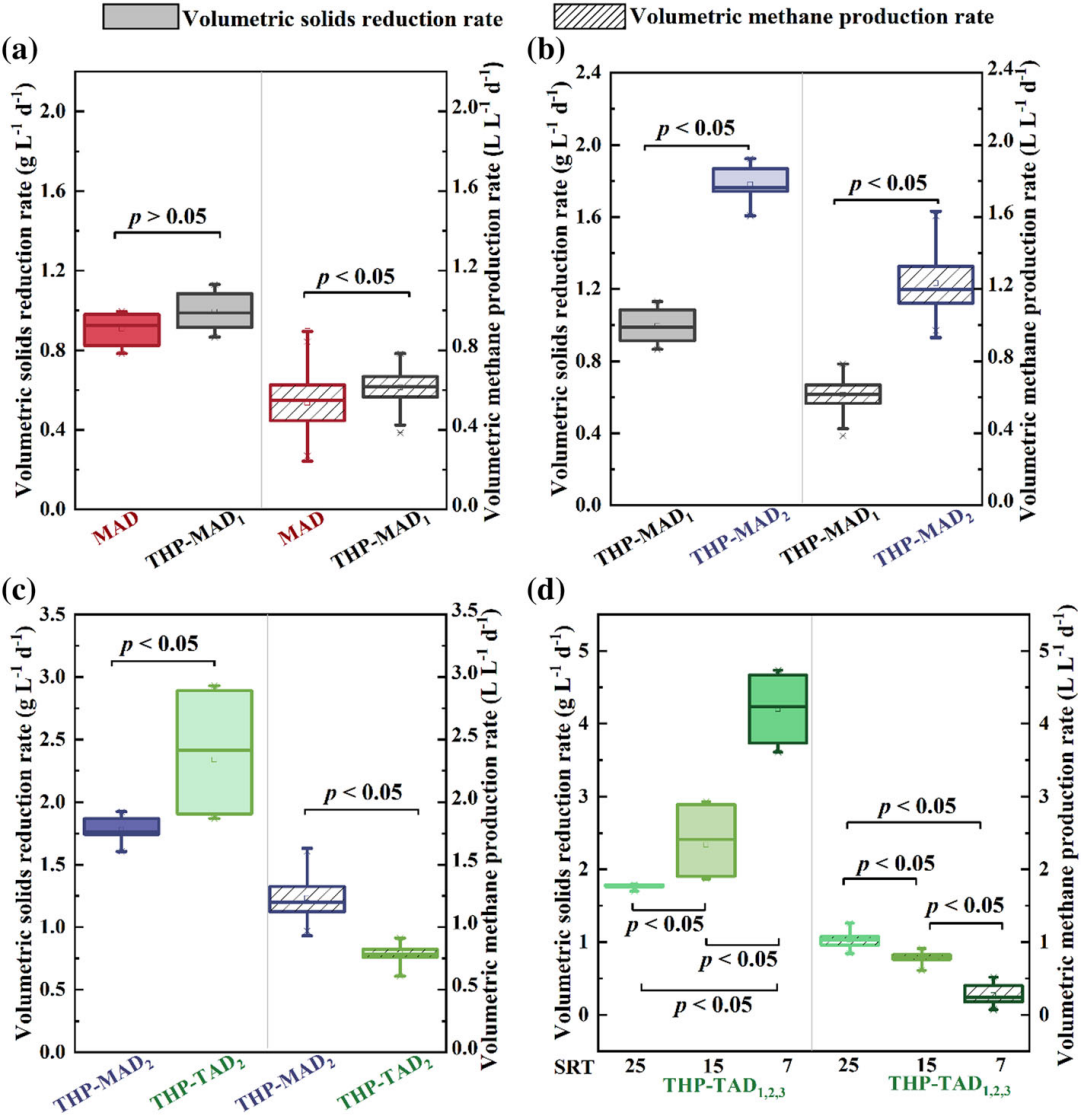
Box plots showing the comparative results of volumetric solid reduction rates and volumetric methane production rates of systems under various experimental design in Figure 1c, that is, (a) MAD versus THP‐MAD_1_; (b) THP‐MAD_1_ versus THP‐MAD_2_; (c) THP‐MAD_2_ versus THP‐TAD_2_; (d) THP‐TAD_1_ versus THP‐TAD_2_ versus THP‐TAD_3_. Data under steady states of all systems (Figure 3) were used except for THP‐TAD_3_, which did not have a steady state. The data from THP‐TAD_3_ were all included.

The 2.5 times greater VFA availability in the effluent of THP (Table 1) was expected to grow more methanogens in the THP‐MAD_1_, which can be reflected by its significantly greater volumetric methane production rate over that of the MAD without THP measured at the steady state (Table 2 and Figure 2a). In contrast, an insignificant difference of volumetric solids reduction rate between MAD and THP‐MAD_1_ was measured in Table 2 and Figure 2a, suggesting that the microbial hydrolysis in MAD can be as good as that of the THP at such a low TS content of 4%. This conclusion can be supported by the similar VSR values measured in the two digesters (Table 2). It is also noteworthy that not much difference was found in the VFA‐to‐alkalinity ratio between MAD and THP‐MAD_1_ at the steady state either (Table 2). Because VFAs are the intermediate products between hydrolysis and methanogenesis assuming acidification and acetification are not the rate‐limiting steps, this VFA‐to‐alkalinity ratio can be used an indicator of whether methanogenesis rate can catch up with the hydrolysis rate because the ratio would have substantially increased, and the pH would have dropped if otherwise (Lossie & Pütz, 2008). The similar VFA concentrations and neutral pH values in the two digesters' effluent suggest that the methanogenesis has paced well with the hydrolysis with or without THP. Again, the reason might have to do with the low TS loaded into both MADs because microbial hydrolysis apparently has handled such a low TS as well as the THP did. This hypothesis was tested in the subsequent TS effect study.

**TABLE 2:** Performance of anaerobic digesters under various scenarios

	MAD	THP‐MAD_1_	THP‐MAD_2_	THP‐TAD_1_	THP‐TAD_2_	THP‐TAD_3_
SRT (days)	15	15	15	25	15	7
Feedstock TS (%)	4.0 ± 0.1	4.0 ± 0.1	8.0 ± 0.1	8.0 ± 0.1	8.0 ± 0.1	8.0 ± 0.1
Feedstock VS (%)	85.0 ± 0.2	85.0 ± 0.2	85.0 ± 0.2	85.0 ± 0.2	85.0 ± 0.2	85.0 ± 0.2
Volumetric solids reduction rate (g L^−1^ d^−1^)	0.91 ± 0.09	0.99 ± 0.10	1.78 ± 0.12	1.76 ± 0.04	2.34 ± 0.46	4.20 ± 0.55
Volumetric methane production rate (L L^−1^ d^−1^)	0.54 ± 0.13	0.61 ± 0.08	1.23 ± 0.15	1.03 ± 0.10	0.80 ± 0.07	0.28 ± 0.12
VSR (%)	46.8 ± 3.7	50.7 ± 4.2	44.1 ± 2.3	63.4 ± 1.1	51.2 ± 7.3	41.0 ± 4.5
VFAs (mg Acetate L^−1^)	1237.5 ± 317.5	1228.1 ± 398.4	3849.6 ± 332.7	4031.5 ± 110.5	5733.6 ± 224.4	7303.9 ± 273.9
Alkalinity (mg CaCO_3_ L^−1^)	4183.2 ± 329.4	4319.1 ± 427.4	6212.3 ± 549.1	6287.2 ± 499.3	5048.8 ± 818.8	4188.7 ± 781.6
VFA‐to‐alkalinity ratio	0.25 ± 0.02	0.24 ± 0.02	0.62 ± 0.04	0.80 ± 0.16	0.96 ± 0.09	2.20 ± 0.34
pH	7.16 ± 0.08	7.16 ± 0.06	7.33 ± 0.07	7.75 ± 0.07	7.66 ± 0.06	6.88 ± 0.25
CH_4_ content in biogas (%)	60.8 ± 1.4	60.1 ± 1.6	57.7 ± 0.50	57.7 ± 0.3	63.3 ± 0.2	20.2 ± 0.1
TAN (mg L^−1^)	698 ± 3	1380 ± 42	1538 ± 3	1960 ± 10	1750 ± 10	1570 ± 10
FAN (mg L^−1^)	11.0 ± 0.1	26.8 ± 0.8	35.1 ± 0.1	349.4 ± 2.2	276.9 ± 1.9	155.4 ± 1.5

*Note*: Data under steady state from all systems (Figure 3) were used except for THP‐TAD_3_. The data from THP‐TAD_3_ were all included.

Abbreviations: FAN, free ammonia nitrogen; MAD, mesophilic anaerobic digester; SRT, solids retention time; TAD, thermophilic anaerobic digester; THP, thermal hydrolysis pretreatment; TS, total solids concentration; VFA, volatile fatty acid; VS, volatile solids concentration; VSR, volatile solid reduction.

**FIGURE 3:**
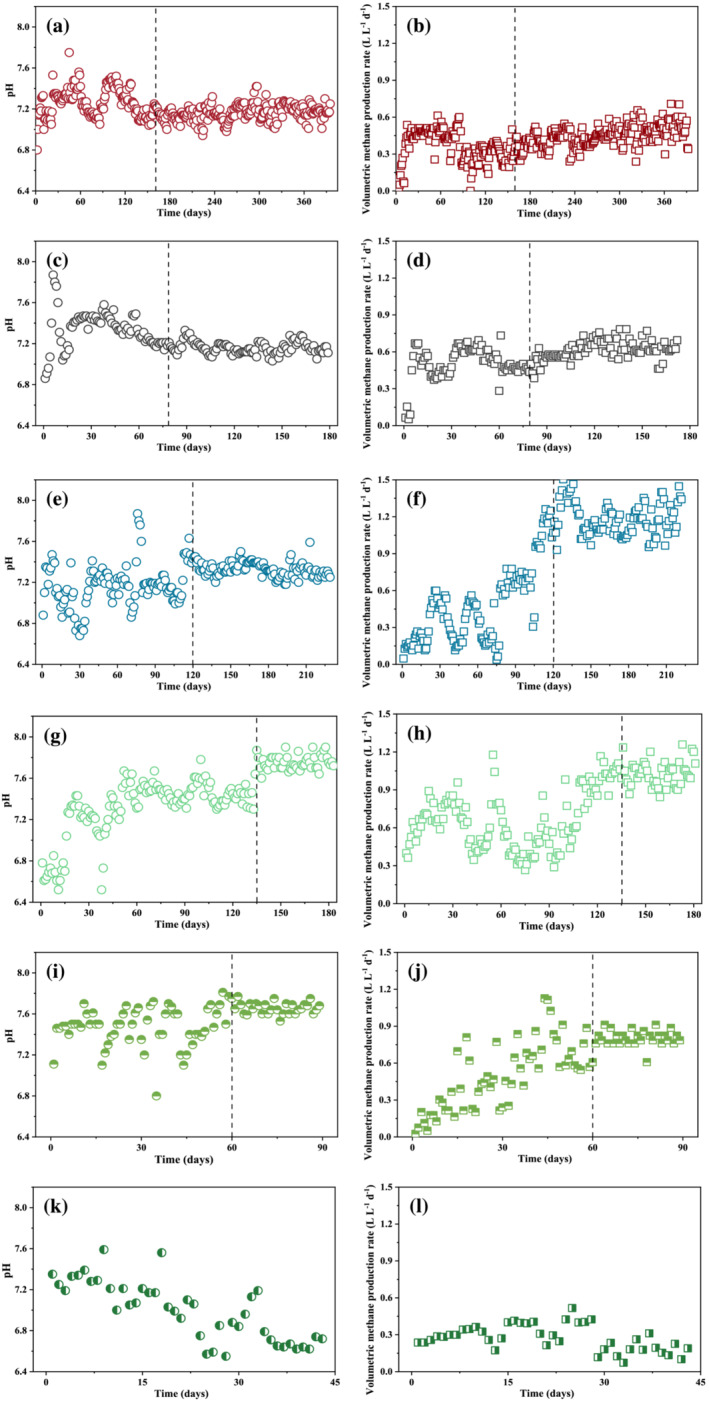
Profiles of pH and volumetric methane production rates for systems under various experimental design described in Figure 1c, that is, (a, b) MAD; (c, d) THP‐MAD_1_; (e, f) THP‐MAD_2_; (g, h) THP‐TAD_1_; (i, j) THP‐TAD_2_; and (k, l) THP‐TAD_3_. Date after dash lines represents data under steady states as determined from *T* test.

### Effects of TS on sludge hydrolysis

The TS that can be effectively mixed via biogas recirculation in these lab‐scale anaerobic digesters is below 8% (Figure 1b). Therefore, two MADs fed with 4% and 8% TS sludge were compared side‐by‐side to understand the TS effect on sludge hydrolysis when all other parameters such as THP, SRTs, and AD temperatures were kept the same. Results in Table 2 show that the VSR declined for 13.0% as a result of doubling the TS in the feed sludge. As a matter of fact, it is expected to see this hydrolysis efficiency decrease because the solids loading applied on the THP‐MAD_2_ has doubled that of the THP‐MAD_1_. Accordingly, the volumetric solids reduction rate and volumetric methane production rate with solids loading rates factored in THP‐MAD_2_ have also doubled those of the THP‐MAD_1_ operated with half solids loading (Table 2 and Figure 2b). This proportional increase in hydrolysis and methanogenesis rates along with the solids loading rates increase suggests that the methanogenesis was still able to keep up with the pace of the hydrolysis increase along with the TS increase and was subjected to minor inhibition from the accumulation of the intermediate products such as VFAs and FAN. Results in Table 2 show that the VFAs and FAN did increase from 1228.1 mg Acetate L^−1^ and 26.8 mg L^−1^ to 3849.6 mg Acetate L^−1^ and 35.1 mg L^−1^, respectively, as a result of doubling the influent TS. The uncompromised rates of solids hydrolysis and methane production just revealed that these levels of intermediate product accumulation were not severe enough to impede the high‐TS MAD performance and thus should be pursued in full‐scale application for better use of the MAD volumetric capacity.

### Effects of AD temperature on sludge hydrolysis

To understand the effects of AD temperature on the sludge hydrolysis, an MAD and a TAD were operated with an SRT of 15 days in parallel with all other parameters kept the same. Results in Table 2 show that the VSR has substantially increased from 44.1% in THP‐MAD_2_ to 51.2% in THP‐TAD_2_ as a result of the AD temperature increase from 35°C to 55°C, highlighting the essential role of AD temperature in governing the sludge hydrolysis rate even with THP applied. As a result of this microbial hydrolysis enhancement, the volumetric solids reduction rate has significantly increased for 31.5% in THP‐TAD_2_ over that of the THP‐MAD_2_ (Figure 2c); however, the volumetric methane production rate has dropped for 53.8% in the THP‐TAD_2_ over that in the THP‐MAD_2_ (Figure 2c). This sharp decline in the volumetric methanogenesis rate has to do with the inhibition from the intermediate‐product accumulation. Table 2 shows that both the VFA‐to‐alkalinity ratio and FAN have substantially increased for 54.8% and 689.1% in THP‐TAD_2_ over THP‐MAD_2_, respectively. Obviously, this intermediate product accumulation can be explained by the inability of methanogenesis to keep up with the pace of hydrolysis in THP‐TAD_2_. The fact that TAD has been used for municipal sludge stabilization for decades without the intermediate product accumulation problem implies that this out‐of‐the‐pace between hydrolysis and methanogenesis is due to the THP integration (Figure 1a). A literature review in Table 3 shows that the volumetric methane production rates have been low in all previous THP‐TAD integrations even though the VSR has been high, indicating that an integration of two high‐rate processes such as THP and TAD has accelerated the hydrolysis rate to the extent that the methanogenesis cannot keep up with anymore. As a consequence, the VFAs and FAN of THP‐TAD_2_ have been dramatically accumulated to the levels of 5733.6 mg Acetate L^−1^ and 276.9 mg L^−1^, respectively (Table 2). Therefore, feeding 8% TS of THP‐processed sludge to a TAD operated at a 15‐day SRT is not a very good choice for methane production even though doing so can substantially improve the sludge hydrolysis performance in terms of VSR and volumetric solids reduction rate as compared with the THP‐MAD_2_ operated under the same conditions (Table 2 and Figure 2c).

**TABLE 3:** Comparison of THP‐TAD performance between previous and this study

Reactor configuration	SRT (Days)	THP(°C)	OLR(kg VS m^−3^·d^−1^)	TS feed(%)	TSR(%)	VSR(%)	Volumetric methane production rate (L L^−1^ day^−1^)	References
Batch	32	134	ND	4.4	ND	ND	0.03	Climent et al. (2007)
Batch	ND	90	ND	8.0	ND	ND	0.13	Yao et al. (2016)
Continuous	10	70	ND	5.4	31.2	36.6	0.61	Ferrer et al. (2009)
Continuous	15	160	3.50	5.7	54.7	60.4	0.95	Han et al. (2017)
Continuous	15	134	1.00	2.1	ND	46.0	0.22	Gianico et al. (2013)
Continuous	15	165–170	3.35	8.3	ND	ND	0.74	Chen et al. (2020)
Continuous	25	165–170	1.88	6.8	ND	ND	0.66	Chen et al. (2020)
Semi‐continuous	25	170	2.61	8.0	55	62.0	1.03	This study
Semi‐continuous	15	170	4.34	8.0	48	53.0	0.80	This study

Abbreviations: ND, not determined; SRT, solids retention time; TAD, thermophilic anaerobic digester; THP, thermal hydrolysis pretreatment; TS, total solids concentration; VS, volatile solids concentration; VSR, volatile solid reduction.

### Effects of SRTs on THP‐TAD

Knowing the THP‐TAD_2_ operation at a 15‐day SRT has already increased the hydrolysis rate to the extent that methanogenesis rate cannot keep up with anymore, the SRT was investigated as a single variable in this experiment to understand whether it can be manipulated to coordinate the paces between hydrolysis and methanogenesis. Theoretically, increasing SRT allows more methanogens to be enriched and thus offers higher methanogenesis rate. Meanwhile, increasing SRT should also enhance VSR for the longer digestion time and thus produce lower TS in the digester effluent. This prediction is in line with the experimental results measured in Table 2; that is, increasing the SRT of TAD from 7 to 15 and then to 25 days has significantly improved the volumetric methane production rates from 0.28 to 0.80 and then to 1.03 L L^−1^ day^−1^ as a result of the more methanogen retention with the SRT increase (Figure 2d). Likewise, the VSR also increased from 41.0, to 51.2, and then to 63.4% along with the SRT increase (Table 2). It is noteworthy that the volumetric solids reduction rate actually decreased along with this VSR increase, and such a VSR increase was proportional to the decrease of volumetric solids reduction rate (Table 2). This can be explained by the first‐order microbial hydrolysis rate that has been recognized in AD (Batstone et al., 2002); that is, the solid hydrolysis rate is supposed to be proportional to the in situ TS in the AD. Hence, a high VSR has attenuated the in situ TS to such a low level that the volumetric solids reduction rate proportional to it per the first‐order reaction kinetics has also become low (Batstone et al., 2002).

It is noteworthy that the volumetric methane production rate in the THP‐TAD_2_ operated at a 15‐day SRT was still significantly lower than that in the THP‐MAD_2_ operated at the same SRT despite the significantly higher volumetric hydrolysis achieved in the former (Figure 2c). Results in Table 2 show that the VFA‐to‐alkalinity ratio and FAN in the THP‐TAD_2_ were both substantially greater than those in the THP‐MAD_2_, indicating that the inhibitor accumulation might be the reason responsible for compromised volumetric methane production in THP‐TAD_2_. This inference is supported by the significantly improved volumetric methane production rate in the THP‐TAD_1_ operated at an SRT of 25 days because both the VFA level and the VFA‐to‐Alkalinity ratio have declined for 29.7% and 16.7% (Table 2) with the SRT increase as more time has been allowed for methanogens to convert more VFAs to methane gas. Yet, the volumetric methane production rate achieved at such a long SRT (25 days) in THP‐TAD_1_ still did not exceed that in the THP‐MAD_2_ operated at a much shorter SRT of 15 days. The ultimate inhibitory factor has to do with the FAN as it is an end product of AD and thus cannot be removed through methanogenesis like VFAs do. FAN inhibition levels to TAD were reported to vary from 45 to 297 mg L^−1^ depending on the microbial communities and acclimation (Capson‐Tojo et al., 2020; Rajagopal et al., 2013). The FAN level of 349.4 mg L^−1^ measured in the THP‐TAD_1_ apparently has exceeded this inhibitory range as a result of the greatest VSR of 63.4% achieved (Table 2). This result just suggested that the FAN inhibition might be the ultimate bottleneck limiting the AD capacity when the rate limitation on the sludge hydrolysis is unblocked.

## DISCUSSION

Judging from the horizontal comparison in Table 2 and Figure 2, the best setup for achieving maximum VSR, namely, 63%, is THP‐TAD_1_ operated at an SRT of 25 days, which is expected in that all three factors; that is, the high temperatures of both AD and THP and the longest SRT, for example, 25 days, have collectively contributed to the solid hydrolysis in this one combination. Meanwhile, the volumetric solids reduction rate of this setup was also comparable with that achieved in the THP‐MAD_2_ operated at an SRT of 15 days, which is the most common setup used across the world (Labatut & Pronto, 2018). The 16.3% lower volumetric methane production rate has to do with the 10 times FAN accumulation as a result of the 55°C used as well as the greatest VSR and pH values measured in the THP‐TAD_1_. This is because FAN with a concentration greater than 297 mg L^−1^ has been shown inhibitory to methanogenesis (Capson‐Tojo et al., 2020; Rajagopal et al., 2013). As mentioned above, FAN is an end product of AD that cannot be reduced by AD itself unless a separate TAN removal process is incorporated. Besides, FAN level is actually very sensitive to the AD temperature used. According to the ideal equilibrium equation (Emerson et al., 1975), an increase of AD temperature from 35°C to 55°C will increase the FAN level for three times even when the pH and TAN remain at the same level of 7 and 1000 mg L^−1^, respectively. With this FAN inhibition in mind, it is not difficult to understand the methanogenesis rate retardation and, in turn, the elevated VFA accumulation (Figure 2d and Table 2) in face of the accelerated hydrolysis rates brought by THP and TAD. As a consequence, both the VFA concentration and the VFA‐to‐alkalinity ratio became higher in all THP‐TAD than those in THP‐MAD as a result of the methanogenesis inhibition (Table 2), causing a feedback inhibition loop between FAN and VFAs as described by Capson‐Tojo et al. (2020). Given the exceptionally higher VSR and VFA production obtained in THP‐TAD over THP‐MAD, the THP‐TAD setup might be more conducive to the applications that emphasize more on the solid removal or VFA recovery than on the methane production because the high temperatures of THP and TAD have improved the hydrolysis so well that the methanogenesis can no longer keep up with anymore.

According to the U.S. Environmental Protection Agency (USEPA) reports, out of the 1484 municipal wastewater treatment facilities that digest municipal sludge to produce biogas in the United States, only less than 10% commercially utilize biogas, leaving the rest just flaring the biogas into the atmosphere or merely combust biogas in boilers (Naik‐Dhungel, 2010). Meanwhile, VFAs are an important carbon substrate to assist mainstream enhanced biological phosphorus removal (EBPR) and biological nutrient removal (BNR) (Atasoy et al., 2018; Luo et al., 2019). Technically, VFAs accumulated in TAD effluent can be recovered and utilized through filtrate recirculation (Yesil et al., 2021). Many studies have shown that better EBPR performance was obtained using VFAs derived from the fermenter than dosing equivalent amounts of synthetic acetic acid (Atasoy et al., 2018; Luo et al., 2019). Meanwhile, 31.5% more solids can be further reduced in a unit reactor volume on a daily basis when using the configuration of THP‐TAD at SRT of 15 days (THP‐TAD_2_) than THP‐MAD_2_ at the same SRT (Table 2). The FAN inhibitory problem might be addressable with the application of in situ ammonia recovery techniques currently under development. Example technologies include in situ ammonia stripping using either biogas (Bi et al., 2020; Walker et al., 2011) or nitrogen (Yao et al., 2017) as stripping gas without pH or temperature modification. Palakodeti et al. (2021) and Zhang et al. (2020) have also reported that adding biochar into continuous or semi‐continuous digesters can not only alleviate FAN inhibition but also enhance digestate fertilizer values. Shi et al. (2019) even demonstrated that submerging a hydrophobic gas‐permeable expanded polytetrafluoroethylene (ePTFE) membrane tube into the AD reactor and recirculating an adsorption solution of sulfuric acid through the membrane tube can effectively achieve in situ and real‐time ammonia recovery from AD systems. It is anticipated that the rate‐limiting step of the municipal sludge AD can be further alleviated in the future with the application of similar in situ ammonia removal technologies.

## CONCLUSIONS

The following concluding remarks can be drawn from this study:

- All THP‐TAD investigated in this study can offer equal or faster volumetric solids reduction rates than all the THP‐MAD do.
- FAN inhibition appears to be an ultimate limiting factor constraining the volumetric methane production rate in THP‐TAD.
- THP‐MAD_2_ operated at an SRT of 15 days with 8% TS influent sludge offered the greatest volumetric methane production rate.
- THP‐TAD_1_ operated at an SRT of 25 days with 8% TS influent sludge offered the greatest VSR at a volumetric methane production rate similar to that achieved in THP‐MAD_2_ and thus should be considered for full‐scale application.
- In situ ammonia removal technique should be developed to further unblock the rate‐limiting step of THP‐TAD_1_.

## AUTHOR CONTRIBUTIONS

**Jiefu Wang:** Data curation; investigation; methodology. **Yuepeng Sun**: Data curation; investigation. **Dian Zhang:** Data curation; investigation. **Tom Broderick:** Conceptualization; funding acquisition; project administration. **Mary Strawn:** Conceptualization; funding acquisition; project administration. **Hari Santha:** Conceptualization; project administration. **Karen Pallansch:** Conceptualization; funding acquisition; project administration. **Allison Deines:** Conceptualization; funding acquisition; project administration. **Zhi‐wu Wang:** Conceptualization; funding acquisition; project administration.

## Data Availability

The data that support the findings of this study are available on request from the corresponding author. The data are not publicly available due to privacy or ethical restrictions.
